# Professor Arlt

**Published:** 1887-05

**Authors:** 


### ARLT.

PROFESSOR ARLT, of Vienna, recently died in that city. He was one of the most eminent scientific ophthalmologists of his day, and at one time enjoyed the reputation of being the most skillful operator upon the eye in the medical profession. His contributions to ophthalmology, both as a teacher and a writer, have been of great value.

